# Supplementary figures and images for: Burst firing creates an attractor in synaptic weight dynamics
Source: PLoS Comput Biol. 2026 Mar 9;22(3):e1014001. doi: 10.1371/journal.pcbi.1014001 (PMC13108889; doi:10.1371/journal.pcbi.1014001)

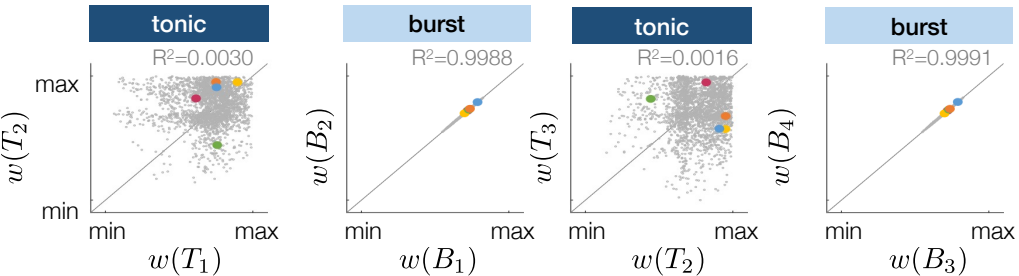

Supplement: S1 Fig — (PDF) [file pcbi.1014001.s002.pdf]

Soft-bounds

Hard-bounds

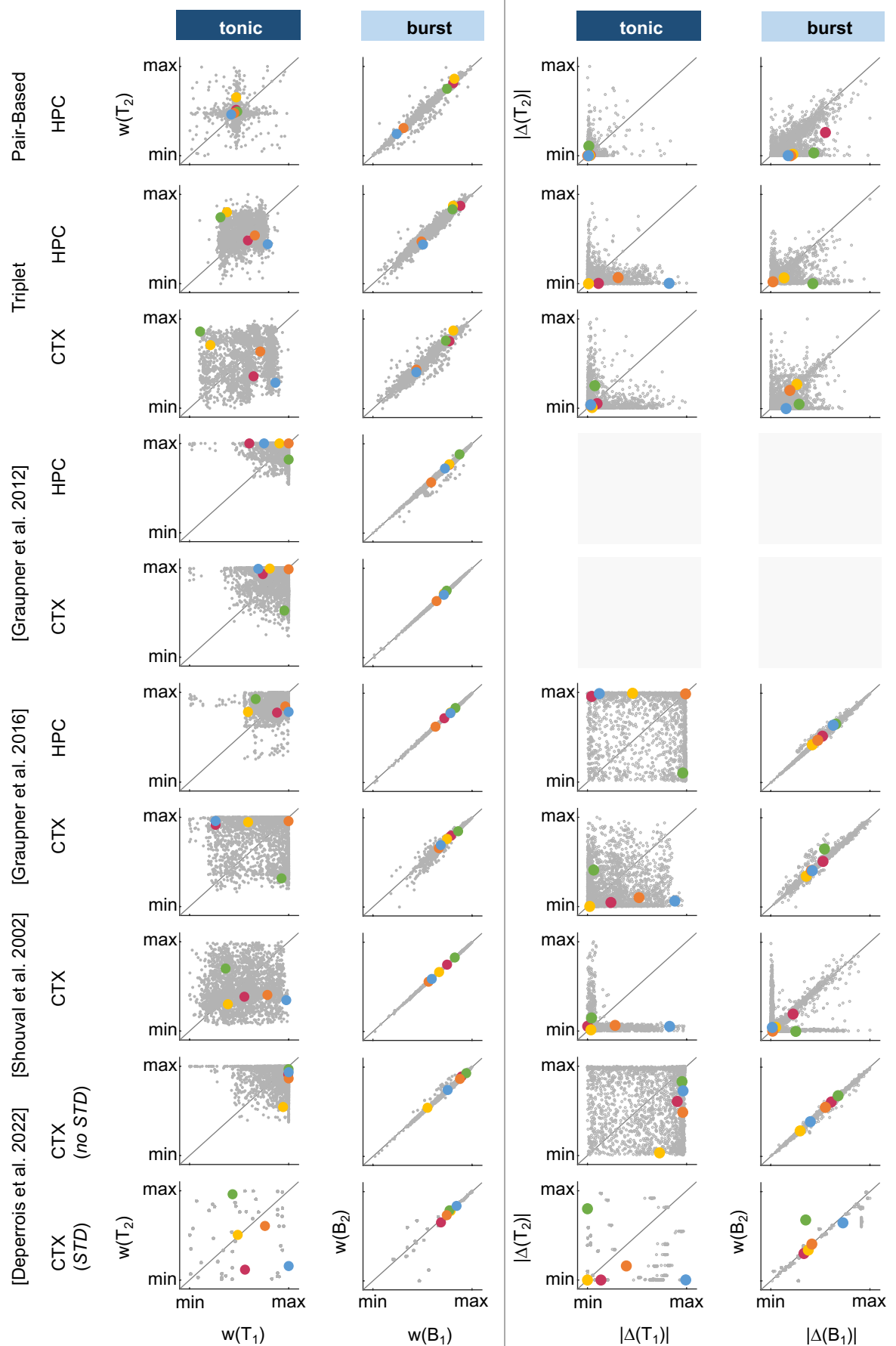

Supplement: S2 Fig — Comparison of the synaptic weights at the end of the third and fourth tonic firing states (left column) or the third and fourth burst firing states (right column), normalized between the minimal and maximal values. (CTX = cortex, data fitted on [35]; HPC = hippocampus, data fitted on [38]). (PDF) [file pcbi.1014001.s003.pdf]

**A**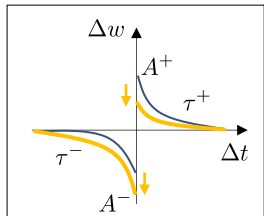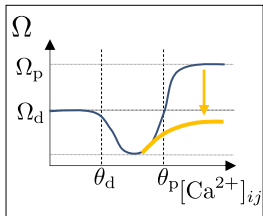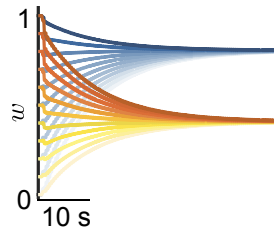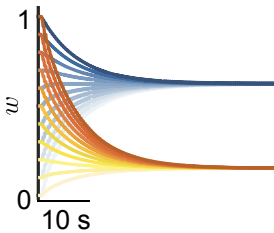**B**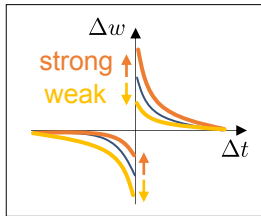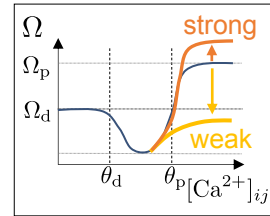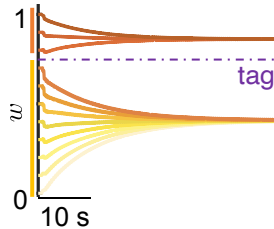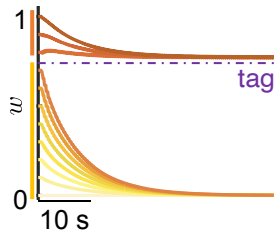

Supplement: S3 Fig — A. Global neuromodulation. Left: plasticity rules in spike-based (top) and calcium-based (bottom) formulations, where potentiation and depression parameters (Ap,Am, or Ωp, Ωd) are globally downscaled (yellow arrows). Right: synaptic weights w converge toward a lower attractor during bursting, showing that global parameter changes shift the attractor in weight space. B. Tag-dependent modulation. Left: plasticity rules where synapses above a tagging threshold receive enhanced potentiation parameters, while weaker synapses are downscaled. Right: tagged synapses converge to a higher attractor (purple dashed line), while untagged synapses decay toward a lower one, resulting in bimodal consolidation. This mechanism shows how neuromodulation and tagging can selectively stabilize strong synapses while weakening weaker ones. This figure shows the evolution of synaptic weights between two excitatory neurons during burst firing under different initial conditions (0:0.1:1). In blue, trajectories correspond to unmodulated plasticity parameters, while in yellow they show neuromodulated parameters. The color gradient emphasizes the initial strength of the synaptic weights, with darker shades indicating larger initial values. (PDF) [file pcbi.1014001.s004.pdf]
