# Supplementary material for: Burst firing creates an attractor in synaptic weight dynamics
Source: PLoS Comput Biol. 2026 Mar 9;22(3):e1014001. doi: 10.1371/journal.pcbi.1014001 (PMC13108889; doi:10.1371/journal.pcbi.1014001)
Supplement: Attachment — Submitted filename: Response to the Editor and to the Reviewers.pdf [file pcbi.1014001.s005.pdf]

Response to the Editor and to the Reviewers

We would like to sincerely thank both the editor and the reviewers for their thoughtful, constructive, and intellectually engaging comments. The different perspectives raised during the review process were genuinely inspiring and encouraged us to reflect on aspects of the project in ways we had not previously considered. While the publication process can often be challenging, this exchange exemplified the good of peer review: a shared curiosity about how neural systems work, a commitment to clarity and rigor, and a collaborative effort to improve the scientific contribution. We are grateful for this interaction and believe it has significantly strengthened the manuscript. Below, we provide our responses to the different sections.

Editor

This work presents a study on tonic and burst firing neuronal dynamics. To strengthen the manuscript, the authors should provide a more detailed justification for the model's assumptions and a more rigorous validation of its dynamics against experimental data, at least by providing more discussions.

We thank the editor for this helpful suggestion. The goal of the present work is to develop a conceptual and computational framework for understanding how synaptic plasticity operates in neural circuits that express multiple firing regimes, rather than to reproduce the dynamics of a specific experimental preparation.

In the revised manuscript, we have expanded the Discussion to clarify the motivation for the modeling assumptions and to place the network dynamics in a broader experimental context. The tonic and burst firing regimes considered here correspond to well-established activity modes observed across many systems, including cortex, thalamus, hippocampus, central pattern generators, and invertebrate circuits. The use of a minimal inhibitory control element reflects a coarse-grained representation of state-dependent modulation, capturing how changes in inhibition or neuromodulatory tone can shift population activity between regimes.

We further discuss how the burst-induced attractor identified in the model generates qualitative predictions that are consistent with experimental observations, such as synchronization during bursts, state-dependent reshaping of synaptic strengths, and neuromodulatory control of consolidation. Importantly, this framework makes testable predictions that could be examined experimentally in a variety of systems, including genetically accessible models such as *Drosophila*, where firing state transitions and plasticity rules can be probed and manipulated in vivo.

We believe that framing this work as a general computational framework, together with an expanded discussion of its biological relevance and experimental testability, strengthens the manuscript and clarifies its scope.

| Corrections in the text (tracked version): lines 331–338, 367-370

Reviewer #1

Major comment: Role of alternation vs. burst regime itself

The major question I would raise is whether this work in fact addresses the dynamics arising from SWITCHING between firing regimes, as claimed. The paper states that plasticity has previously "been studied only in either one of these regimes," and that this study seeks to clarify how ALTERNATING between tonic and burst firing shapes long-term synaptic weight dynamics. However, it is not entirely clear that the ALTERNATION itself contributes new dynamical structure beyond what is already observed in each regime separately.

The model convincingly shows that tonic firing produces heterogeneous, broad weight distributions, and burst firing produces homogeneous, convergent weight states. Yet when these regimes are concatenated in alternation, the resulting dynamics appear to be merely the superposition of two independent phases: the weight evolution during each phase depends only on the instantaneous plasticity rule and activity statistics, not on the prior regime. Consequently, in the baseline model, the tonic phase has no causal influence on the subsequent burst phase (and vice versa). Only when additional mechanisms such as synaptic tagging or parameter modulation are introduced does the alternation acquire genuine dynamical significance. However, it could be argued that even these effects do not strictly require an explicit tonic phase: one could equivalently initialise the system with a (heterogeneous) distribution of weights, assign pre-tagged synapses/neuromodulation, and then evolve it under the burst-regime dynamics to obtain the same attractor differentiation. In that sense, the alternation between regimes is not essential for demonstrating the key mechanism; rather, it provides a plausible biological context for how such conditions might arise. I acknowledge that this contextual role is important, as the suggested link with possible learning and memory mechanisms (such as sensory processing, memory consolidation, and a "reset") rests conceptually on the alternation between regimes. Pointing out the distinction between the model's dynamical necessity and its biological interpretation would clarify the paper's central claim.

I would maybe suggest a slight reframing to emphasise that the alternation between regimes is what enables the proposed functional interpretations - linking distinct dynamical regimes to phases of learning and memory - rather than primary driver of distinct weight dynamics. In this view, the alternation provides the biological and computational context that makes these mechanisms relevant, even if the underlying plasticity dynamics arise locally within each regime.

We thank the reviewer for this insightful perspective, which motivated us to refine the framing of the role played by alternation between tonic and burst firing. We agree that the burst-induced attractor arises from the plasticity dynamics intrinsic to the burst firing regime itself and does not depend on alternation per se: for instance, similar convergence would be observed starting from random initial weights in isolated burst states, provided plasticity parameters are fixed.

However, alternation between tonic and burst firing reflects the physiological operating conditions of neural circuits and provides the biological and computational context that makes these dynamics functionally relevant. Tonic firing supports input-driven learning and leads to

heterogeneous synaptic configurations, whereas subsequent burst firing reorganizes these configurations by driving weights toward a convergent attractor. In this sense, alternation sequences, rather than fundamentally changing the plasticity dynamics associated with each regime, structure when these dynamics are engaged and how they can be interpreted in terms of learning and consolidation.

We have revised the manuscript to make this distinction explicit, emphasizing that alternation provides a temporal scaffold linking regime-specific plasticity dynamics to phases of learning and memory, rather than acting as the primary driver of synaptic weight dynamics. We believe this clarification strengthens the conceptual scope and interpretability of the results.

| Corrections in the text (tracked version): lines 26, 41, 55-58, 121-125, 367-368; Abstract, Author Summary, and relevant figure captions

Minor comment: Relationship between bursting activity and external input current

Just something I want to clarify: on lines 157 to 159 it is stated that the bursting activity "is independent of external input currents $I_{\text{app},j}$ and $I_{\text{app},i}$", but in Fig1B, $I_{\text{app},j}$ is off/silent. It is not fully clear whether bursting would remain independent if $I_{\text{app},j}$ continued to deliver pulses, as in the tonic phase. Methods describe $I_{\text{app},i}$ as fluctuating between 0 and 1 nA/cm2, but this does not say anything about the independence of the bursting activity from input. Currently, the figure demonstrates independence from SILENCE, but not from ACTIVE INPUT. It is only a minor clarification, and would not impact the results or conclusions.

By “independent of external input currents,” we did not intend to imply that bursting is invariant to any conceivable input, but rather that, in the regime studied here, rhythmic bursting is generated endogenously by intrinsic neuronal and network mechanisms, rather than being driven or paced by structured external stimulation. This point builds on our previous work (Jacquerie et al., 2021), which demonstrated both the mechanism and robustness of endogenous burst firing in this class of network models.

In Fig. 1B, external pulse-train inputs are silenced to illustrate this: once the inhibitory drive is switched on by hyperpolarizing the inhibitory neuron, collective bursting emerges without requiring patterned external input. During burst firing, the small fluctuating baseline current described in the Methods serves only as background noise and does not impose temporal structure on the activity. Importantly, the amplitude of this baseline current is far below that required to elicit action potentials during tonic firing, where substantially larger depolarizing inputs are needed to reach spike threshold.

We draw this distinction to emphasize the distinction from previous modeling studies in which burst firing is imposed externally, for example by square-wave depolarizing currents that

alternate between silence and tonic firing. This is not the case here. In our model, bursting arises from intrinsic ion channel dynamics—most notably low-threshold calcium currents—that are unmasked by changes in inhibition, making the bursting activity genuinely endogenous. We agree that external inputs at sufficient amplitude or with specific temporal structure could alter or entrain the bursting pattern; this possibility is outside the scope of the present study but does not affect the emergence of the endogenous burst regime analyzed here.

We have clarified this distinction in the text to avoid ambiguity. As the reviewer notes, this clarification does not affect the results or conclusions of the study.

| Corrections in the text (tracked version): lines 93-97, 367-369, 412-419, 430-433

R^2 in the tonic regime & difference of timescales during tonic and burst firing

In section 4.5 it is mentioned that the R^2 in tonic firing is lower than in burst firing due to "finite-time effects" (i.e. "some synapses are predicted to depress toward zero but have not yet completed that trajectory within the 50 s window, leading to larger residuals"). This raises a practical question: Why not extend the window to reduce the finite time effects? How long would you need to simulate tonic firing to achieve comparable convergence to burst firing? Also, the authors could consider the fact that maybe the noise level/stochasticity of the tonic phase also contributes (a lot?) to the lower R^2; this might be worth adding.

We thank the reviewer for raising these points. The lower R^2 observed during tonic firing reflects both finite-time effects and the fundamentally weaker convergent structure of the tonic regime. While extending the tonic firing duration would indeed reduce finite-time residuals, achieving convergence comparable to burst firing would require substantially longer simulations—on the order of several hundreds of seconds to minutes for many synapses—because tonic firing provides weak, noisy, and largely uncorrelated plasticity drive.

In tonic firing, each synapse is driven by its own stochastic input spike train, leading to heterogeneous calcium dynamics and large variability in the effective time spent above plasticity thresholds. As a result, some synapses approach their analytical fixed points only very slowly, and others may remain effectively inactive for long periods. Increasing simulation duration would reduce variance but would not produce the rapid, uniform convergence characteristic of burst firing.

By contrast, collective burst firing synchronizes spiking and calcium dynamics across synapses, generating strong, highly correlated plasticity signals that drive fast and homogeneous convergence toward a well-defined attractor within tens of seconds. Thus, the higher R^2 observed during bursting reflects not only finite-time effects, but a qualitative difference in the strength and structure of synaptic convergence between tonic and burst regimes.

We have clarified this distinction in the revised text by explicitly noting the contributions of both finite-time effects and stochasticity in tonic firing.

Relatedly, the timescales over which the attractor forms could be important for your interpretations/conclusions. Understanding this relationship (e.g. how rates of convergence depend not only on plasticity parameters but also on the initial weight distribution) could provide insight into the functional relevance of bursts, such as how long bursting activity is required to stabilise synapses or how tonic-induced variability shapes consolidation dynamics. Perhaps the authors can comment on whether heterogeneous timescales in attractor formation are present in the model and if this has a bearing on the functional interpretations?

We thank the reviewer for this insightful comment. In our model, the timescale over which synaptic weights converge toward the burst-induced attractor is primarily determined by the plasticity rates and by the degree of synchrony in the plasticity-driving signals, rather than by the initial weight distribution alone. During collective burst firing, neuronal synchrony produces highly similar calcium dynamics or spike-time correlations across synapses, which leads to relatively uniform and rapid convergence toward the attractor on comparable timescales.

In contrast, during tonic firing, convergence times are strongly heterogeneous: synapses experience distinct and noisy input statistics, and some may spend little or no time above plasticity thresholds. As a result, tonic-induced weight evolution is slow and uneven, and effective convergence toward analytical fixed points can require very long durations, if it occurs at all.

Beyond this synchrony effect, transitions into burst firing could also alter the effective timescale of plasticity itself, for example through burst-dependent neuromodulation that rescales plasticity parameters. In such cases, synaptic weights would still converge toward the same burst-induced attractor, but on a different timescale than the one illustrated here. Burst firing may therefore regulate not only where synapses converge in weight space, but also how *fast* consolidation proceeds.

Functionally, these results suggest that tonic firing promotes synaptic variability and exploration, while burst firing acts as a consolidation phase in which synapses are driven efficiently and homogeneously toward stable configurations. Short or repeated burst episodes would move synapses partially toward the attractor, allowing the rate and extent of stabilization to depend on burst structure, neuromodulatory context, and plasticity timescales. We now emphasize these points in the Discussion.

Finally, prompted by the reviewer's comment, we clarify an edge case in the analytical description. When a synapse spends no time in either the potentiation or depression regimes (i.e., calcium remains below both thresholds), the analytical expression for the fixed point is undefined, reflecting the fact that plasticity is inactive. In this case, the synaptic weight remains at its initial weight value at the onset of the firing regime (or state switch). We have explicitly clarified this point in the manuscript.

| Corrections in the text (tracked version): lines 177–184, 210-232, 294-297, 404, 412-419

Prediction of the neuromodulation-induced shift of the attractor

In section 4.6 the authors point out that modulation of external drive can result in the attractor shifting "slightly", depending on the bursting regime. Is there a way of quantifying or making the degree of "shift" more precise? The 3 different attractors in Fig 3A are arguably noticeably different. Being able to quantify the shift would allow the reader to understand how slight or substantial it is, and permit one to measure how sensitive the attractor is to the external current, to understand if the relationship is linear or not.

In our model, varying the external inhibitory drive alters the collective bursting regime of the network. Because neurons are heterogeneous and interact nonlinearly, changing inhibition modifies multiple burst features simultaneously, such as burst timing, spike count, duration, and synchrony, rather than producing a simple scaling of activity across neurons. These changes propagate to calcium dynamics in a nonlinear manner and ultimately shape synaptic weight evolution.

For this reason, although the attractors shown in Fig. 3A are clearly distinct, we cannot define a simple analytical mapping between the external current and the resulting attractor location. The model involves many interacting variables (intrinsic conductances, burst structure, calcium thresholds, and plasticity nonlinearities), so the attractor position cannot be predicted from inhibition level alone. Rather, modifying the global network state reliably reorganizes synaptic weights toward different stable configurations, with the magnitude of the shift depending on the emergent bursting dynamics in each regime.

To nevertheless quantify how the attractor shifts with inhibition, we performed simulations across a range of external inhibitory drive values. For each condition, we measured the value of the synaptic weights at the end of the burst firing states and computed the mean attractor position across realizations; variability across networks is indicated by the error bar (standard deviation). This analysis shows that while attractors corresponding to different bursting regimes are distinct, their positions vary smoothly as a function of inhibitory drive, with modest changes in amplitude over the explored range (update Fig 3B).

| Corrections in the text (tracked version): lines 243-265, Fig3B

Typos
We thank the reviewer for spotting the typos. They have been corrected in the revised manuscript.

<u>Reviewer #2</u>

Timescale of attractor convergence vs. timescales of tonic and burst regimes

An important conceptual question is the time scale of synaptic plasticity is far longer than the switching between tonic and burst. The figures indicate the weights only take few dozens of seconds to reach stationary, which should not be the case in reality. Although I understand this is probably a math simplification to speed up the simulation and doesn't affect the conclusion substantially, it will be important for the author to explicitly explain this assumption. In reality, the time spent of burst firing mode is short, unlike the sustained bursting firing lasting for 20s in the simulation. My understanding of the sustain burst firing in the simulation is only for math analysis. Nevertheless, considering the few moments of burst firing observed in reality, I wonder how much the burst firing play a significant role in shaping learned synaptic weights? It worths some discussions.

We thank the reviewer for raising this important point regarding the apparent mismatch between synaptic plasticity timescales and the duration of burst firing in the simulations. In the present work, tonic and burst firing durations were chosen to allow synaptic dynamics to be examined clearly within a tractable simulation window, rather than to reproduce the exact temporal structure of a specific experimental system.

Importantly, burst firing is not restricted to isolated, millisecond-scale events. Sustained or recurrent burst firing over seconds to tens of seconds has been observed across multiple brain circuits and behavioral states (e.g., Steriade et al., 2001; Lisman, 1997; Hedrick and Waters, 2012; Shao et al., 2021). Thus, the prolonged bursting used in the simulations does not reflect a claim about single burst episodes in vivo, but rather captures regimes in which bursting repeatedly dominates neuronal activity.

The convergence rates observed in the model are determined by the parameters of the plasticity rules themselves, which are taken from previously published and experimentally grounded models. Sustained burst firing is therefore used as a modeling device to reveal the fixed points and attractor structure of synaptic dynamics under a burst-dominated firing regime. Demonstrating convergence under prolonged bursting does not imply that synapses must reach the attractor within a single burst episode. Instead, shorter or intermittent bursts would produce partial shifts toward the same attractor, which could accumulate over repeated episodes or interact with neuromodulatory and slower consolidation processes.

Overall, the present results characterize the organizing effect of burst firing on synaptic dynamics, without making claims about the absolute duration of burst episodes or consolidation times in biological circuits.

| Corrections in the text (tracked version): lines 209-232, 253-265
